# Coordinated regulation of colonic fluid and mucus secretion

**DOI:** 10.3389/fphar.2026.1757501

**Published:** 2026-03-18

**Authors:** Alan W. Baird

**Affiliations:** UCD School of Veterinary Medicine, University College Dublin, Dublin, Ireland

**Keywords:** colon, epithelium, mucus, secretion, water transport

## Abstract

The human colon, unlike the small intestine which is relatively sterile, contains a diverse microbiome which contributes to host metabolism. The luminal environment is constantly changing and responds to epithelial absorption and secretion which occurs as motility is regulated by longitudinal and circular smooth muscle. Mucous gels are crucial to lubrication and maintenance of an unstirred layer which separate the epithelium from the lumen. Gel-forming mucins are produced and released by goblet cells and become hydrated, although the source of water is not definitively understood. The purpose of this review is to summarize regulation of water movements across the colonic epithelium, goblet cell secretion of mucus and to consider how these distinct processes are functionally coupled.

## Introduction

The human colon is the largest section of the large intestine and plays a key role in digestion, eliminating unabsorbed contents and regulating fluid balance (Santucci and Velez, 2024). It starts after the caecum (where the small intestine ends) and extends to the rectum. The anatomy of the colon including its musculature, vasculature, innervation, and lymphoid system is well understood and underpins knowledge of its functions in health and disease (Peterson and Artis, 2014; Odenwald and Turner, 2017; Salim and Soderholm, 2011; Coskun, 2014). Its structure and function has been described as a ‘masterpiece of biological complexity’ (Daniel, 2023). For example, motility, fluid movements (secretion/absorption), and electrolyte homeostasis are regulated by the enteric nervous system (Avetisyan et al., 2015; Furness, 2000; Mayer et al., 2014), the autonomic nervous system (Mayer et al., 2014), the central nervous system (Mayer and Tillisch, 2011; Tait and Sayuk, 2021), along with endocrine controls (Penning et al., 2000) and luminal microbiota (Daniel, 2023; Segers et al., 2019) Effectors through which regulation occurs include epithelial ion channels, aquaporins (Sisto et al., 2019; Zhao et al., 2021) and inflammation (Peterson and Artis, 2014; Salim and Soderholm, 2011). These processes and mechanisms support the colon’s three principal functions: absorption, secretion, and bacterial fermentation.

The colon is an epithelial-lined, muscular, vascularised tube which is embryologically derived from all three germ layers which develop simultaneously during embryogenesis (Kostouros et al., 2020; Noah et al., 2011). The epithelial layer acts as a permissive, dynamic barrier which separates the luminal compartment from the underlying lamina propria. The epithelium is a heterogeneous sheet of cells lining the entire gastrointestinal tract derived from stem cells. The colonic epithelium is arranged as crypts in which stem cells continuously differentiate and migrate towards the luminal surface producing the mature epithelial cell types. Stem cells of the crypt turnover every 3–7 days (Barker, 2014) and are capable of producing all differentiated epithelial cell types (Barker, 2014; Cl and evers, 2013; Barker and Clevers, 2007; Sato et al., 2009).

The predominant epithelial cell type is the colonocyte, responsible for absorbing water and electrolytes from the fluid delivered from the small intestine. The next most numerous cell type are goblet cells especially in the distal colon which secrete mucus for lubrication and barrier function (Vancamelbeke and Vermeire, 2017). Columnar and goblet cells together make up almost 95% of the total epithelial cells (Geibel, 2005). Other colonic epithelial cells (Cleynen and Laukens, 2019) include enteroendocrine cells which secrete humoral factors to coordinate gut function and Paneth cells which secrete antibacterial peptides into the luminal compartment (Johansson and Hansson, 2011; Clevers and Bevins, 2013). Single-cell profiling of colonic epithelial cell types displays a differentiation hierarchy (Parikh et al., 2019; Schumacher, 2023).

The gross anatomy of the colon divides it into sections with some segmental heterogeneity in structure and also in function (Moran and Jackson, 1992; Keely et al., 1995; Carey, 1977; Vadlamudi et al., 2012). The contents of the colon include water, electrolytes, mucus, undigested materials, and faeces. By the time luminal contents arrive at the proximal colon, most digestion and nutrient absorption have occurred leaving undigested fibre, some water and electrolytes. Unlike the small intestine which is largely sterile the human colon contains trillions of microorganisms (Jensen et al., 2023; Sender et al., 2016; Kho and Lal, 2018). During transit of the colon excess water is absorbed and a significant degree of microbial digestion occurs which contributes significantly to metabolism (Livesey et al., 1995; Bryant et al., 2023; Krajmalnik-Brown et al., 2012; McBurney, 1994; McNeil, 1984).

The proximal and distal segments have separate neurovascular supplies which reflect embryological development and also disease susceptibility (Sandle et al., 1986). Transport mechanisms differ along the colon’s length quantitatively and qualitatively with proximal colon involved in initial fluid and nutrient recovery. The pH in proximal colon is relatively low due to the production of short-chain fatty acids by gut bacteria and segmental heterogeneity of the colonic microbiome is also a feature (Flynn et al., 2018; Yang et al., 2025). In spite of their differences, each segment contributes to the shared functions of absorption, secretion, and bacterial fermentation.

## Water movements across the colonic epithelium

The lumen of the colon is largely an aqueous environment. Colonocytes absorb water and electrolytes (Geibel, 2005; Negussie et al., 2022). 1–2 L of fluid per day reaches the colon from the small intestine where absorption maintains hydration and forms stools (Kiela and Ghishan, 2016; Sandle, 1998). Transepithelial water movements follow osmotic gradients set by transepithelial ion transport (Geibel, 2005; Negussie et al., 2022; Keely and Barrett, 2000). The colonic epithelium is regarded as ‘tight’ on account the seal formed by tight junctions, which are protein complexes which join colonic epithelial cells and selectively permit the passage of substances between cells. The epithelial sheet maintains the separation of luminal contents from the lamina propria and generates osmotic, chemical, electrical, hydraulic, and mechanical gradients between the luminal compartment and the subepithelial extracellular domain. Thus the colon is adapted to salvage water by absorption which follows sodium and chloride ion absorption (Kiela and Ghishan, 2016; Goodman, 2002). The colonocytes actively transfer electrolytes from the lumen into the spaces between the cells and water follows through tight junctions between epithelial cells (paracellular route) and/or aquaporins (transcellular) which facilitate transepithelial water movement in colonocytes (Ikarashi et al., 2016; Liao et al., 2021). Tight junctions define the boundary of the apical and basolateral membranes of colonocytes (Farquhar and Palade, 1963; Schmitz et al., 1999) and, along with the mucous layers, are a seminal component of the epithelial barrier (Capaldo et al., 2017).

Colonic epithelial cells maintain a balance between secretion and absorption of fluids and ions. These processes involve membrane proteins such as the cystic fibrosis transmembrane regulator (CFTR), epithelial sodium channels, Na^+^/K^+^/Cl^−^ cotransporters, sodium-hydrogen exchangers, and H^+^/K^+^ATPase. These account for homeostatic regulation of pH modulation as well as the establishment of osmotic gradients which account for both secretory and absorptive water movements (Negussie et al., 2022; Sandle, 1998; Keely and Barrett, 2000) and which also create microenvironments for microbial populations (Yang et al., 2025; Chikina and Matic Vignjevic, 2021). Furthermore, chloride-led water secretion may have a direct effect on bacterial-epithelial interactions since water transport, and associated changes to the mucus gel layer, promotes colonization by lactobacilli, promoting colonic homeostasis. (Musch et al., 2013; Colgan, 2013).

Net water balance in normal colon is absorptive (Negussie et al., 2022; Kiela and Ghishan, 2016). However, colonocytes can be changed from absorptive to secretory states by chloride-led fluid secretion (Keely and Barrett, 2000; Barrett and Keely, 2000; McKay and Perdue, 1993a; McKay and Perdue, 1993b; Cooke, 1994). The molecular basis and regulatory aspects of fluid secretion by the colon are well understood (Barrett and Keely, 2000). Secretion can increase suddenly and dramatically during infections or inflammation and is accounted for by active movements of ions (Geibel, 2005; Negussie et al., 2022; Kiela and Ghishan, 2016; Keely and Barrett, 2000; Barrett and Keely, 2000). Several types of signals including neurotransmitters, hormones. paracrine messengers, inflammatory mediators and toxins act as secretagogues (Geibel, 2005; McKay and Perdue, 1993a; McKay and Perdue, 1993b; Halm, 2004; Specian and Neutra, 1982) (Table 1). That the colon can absorb and also secrete water has implications for mucus hydration, structure, and function. For example, absorption normally prevents excessive water loss in stool. However, fluid loss occurs during diarhoea, including that caused by infectious agents and toxins which lead to loss of body water, with or without salt, at a rate greater than the body can replace it. Diarrhoeal disease is a major global cause of mortality and morbidity (Gottlieb and Heather, 2011). Host responses to parasites which spend some or all of their life cycle in the colon are further examples of functional coordination of water and mucus secretion as effector mechanism to expel parasites (Baird and O'Malley, 1993; Farthing, 2003; Berin et al., 1999).

**TABLE 1:** Although mechanistically uncoupled, colonic fluid secretion and colonic mucus secretion share a wide variety of secretagogues which may account for a level of functional coupling.

Fluid secretion	Mucus secretion
Neurotransmitters (CNS and ENS) (Keely and Barrett, 2000; Barrett and Keely, 2000; McKay and Perdue, 1993a; McKay and Perdue, 1993b; Cooke, 1994; Xue et al., 2007)	Neurotransmitters (CNS and ENS) (Specian and Neutra, 1982; Gustafsson et al., 2012; Ermund et al., 2013a; Specian and Neutra, 1980; Neutra et al., 1982; Phillips, 1992; Specian and Oliver, 1991; Ermund et al., 2013b; Laburthe et al., 1989; Plaisancie et al., 1998; Plaisancie et al., 1994)
Endocrine (Latorre et al., 2016; Wapnir and Teichberg, 2002; Burleigh and Banks, 2007; Chandrasekharan et al., 2013; Luckhoff and Horster, 1984; Moriarty et al., 2001)	Endocrine (Laburthe et al., 1989; Plaisancie et al., 1998; Plaisancie et al., 1994)
Paracrine (Keely et al., 1995; Mawe and Hoffman, 2013; Brown, 1996; Schultheiss et al., 2006)	Paracrine (Johansson and Hansson, 2016; Dharmani et al., 2009)
Inflammatory mediators (Rask-Madsen, 1986; Wallace, 2019; Fujii et al., 2016; Halm and Halm, 2001; Santos and Perdue, 1998)	Inflammatory mediators (Johansson and Hansson, 2016; Garcia et al., 2009; Phillips and Wilson, 1993; Kim and Khan, 2013; Grootjans et al., 2013a; Grootjans et al., 2013b; Wlodarska et al., 2014)
Parasites and Toxins (Wapnir and Teichberg, 2002; Burleigh and Banks, 2007; DuPont, 2009)	Parasites and Toxins (Cornick et al., 2015; Dharmani et al., 2009; Kim and Khan, 2013; Gillois et al., 2018; Sharpe et al., 2018; Moore et al., 1996)

Although mucus and water secretion are usually functionally coupled, this association may be ruptured in pathological circumstances. For example, in cystic fibrosis (CF), the Cystic Fibrosis Transmembrane Conductance Regulator (CFTR) which is a protein-coding gene that acts as an ion channel on cell membranes, including colonic epithelial cells, controlling the movement of chloride and water is functionally impaired. The defective CFTR gene causes colonic mucus build up in several organs including the intestine (Morrison et al., 2019).

## Mucus secretion

The second most numerous cell type are goblet cells colon which form and secrete mucus for lubrication and barrier function (Song et al., 2023; Joja et al., 2025; Paone and Cani, 2020; Bansil and Turner, 2018; Atuma et al., 2001). Mucus consists of mucins which are formed and stored in goblet cells from which mucins are released either constitutively or in response to a secretagogue. Enterocytes contribute to the mucus layer by producing transmembrane mucins such as MUC3, MUC12 and MUC17 and gel-forming mucins, (principally MUC2 in the colon) (Allen et al., 1998). Upon secretion, gel-forming mucins which are highly glycosylated undergo hydration and ion exchange to expand into a gel (Seidler, 2013; Johansson et al., 2013). The mucus layer maintains a reasonably constant depth (Gustafsson et al., 2012; Gustafsson and Hansson, 2025) suggesting that rates of formation and degradation are similar. The role of mucus and mucins has been extensively reviewed (Sheng and Hasnain, 2022; Corfield, 2018; Hattrup and Gendler, 2008; Johansson and Hansson, 2016; Thornton and Sheehan, 2004; Hansson, 2019). Mucus is secreted by goblet cells directly onto the epithelial surface within the GI tract (Atuma et al., 2001), forming a mobile outer layer, that is relatively quickly replaced, and a stationary gel–like layer that is adheres to the luminal surface (Atuma et al., 2001; Johansson et al., 2011).

The colon has a layered mucus system (Atuma et al., 2001; Johansson et al., 2013; Johansson et al., 2011; Johansson and Hansson, 2013). Densely packed mucin polymers form a three-dimensional inner mucus layer. This network of the inner layer results from covalent and hydrogen bonds between glycosylated mucins (Kramer et al., 2015) which self-assemble as mucins and water are co-secreted. As goblet cell secretion goes on, the gel is replaced and migrates away from the epithelial surface, undergoing proteolytic and glycosidic degradation, leading to gel expansion which becomes a less structured, more permeable outer mucus layer (Song et al., 2023). Expansion of mucus gel, which is predominantly water, is driven by the proteolytic cleavage of the cysteine-rich parts of the mucin protein at the C terminus and the capacity of the mucin glycans to bind water (Hoskins and Boulding, 1981; Miller and Hoskins, 1981). The outer mucous layer is colonized by gut microbes, (Martens et al., 2018; Luis and Hansson, 2023), and provide metabolic substrates for commensal microorganisms (Johansson et al., 2011; Miller and Hoskins, 1981).

The role of mucins in forming and maintaining mucous barriers at a range of anatomical sites has been reviewed (Wagner et al., 2018; Kamphuis et al., 2017). As a hydrogel which behaves as a viscous liquid, mucus is composed of large MW glycoproteins (Kang et al., 2022; McGuckin et al., 2011), electrolytes and proteins (Howard et al., 2021; Yang and Yu, 2021). Yet 95%–98% of a mucous gel is water and its biophysical properties including viscoelasticity are dynamic (Johansson and Hansson, 2013).

Goblet cell secretion which leads to the inner layer involves mucin exocytosis which is a Ca^2+^-regulated process which can be classified into two modes - basal or constitutive mucin secretion or stimulated secretion which occurs in response to stimuli (Song et al., 2023; Gustafsson and Hansson, 2025; Cornick et al., 2015) (Table 1). The colonic mucus is protective as a lubricant and also as a component of the innate and acquired immune systems (Sheng and Hasnain, 2022; Corfield, 2018; Pelaseyed et al., 2014). The layers are not uniform and comprised of a relatively dense inner layer and a looser outer layer (Song et al., 2023; Johansson and Hansson, 2016; Johansson et al., 2011).

The mucous gel provides a habitat and nutrients for commensal bacteria in the sacrificial outer layer which transitions from the more tightly packed and organised inner mucus layer which is typically sterile (Kamphuis et al., 2017; Grondin et al., 2020). Complex mucus multilayers have also been observed in a cell culture model using colonic cells grown in an elegant arrayed human *in vitro* 3D crypt construct (Villegas-Novoa et al., 2024). How hydrated mucins maintain their lamellar structure remains to be determined (Round et al., 2012; Meldrum et al., 2018; Nilsson et al., 2014).

Subpopulations of goblet cells have been described (van der Post et al., 2019). These include surface goblet cells which constitutively secrete mucus (Johansson, 2012). In contrast, goblet cells in the upper part of the crypts secrete mucus in response to stimuli such as secretagogues and/or microbial challenge (Birchenough et al., 2015). Thus, basal and stimulated mucus secretion may occur independently although both involve regulation by intracellular calcium. Activation of the calcium signalling pathway in goblet cells induces mucus secretion. The inner mucous layer is constantly renewed by secretions from goblet cells, with a rapid turnover rate that ensures continuous protection (Johansson, 2012; Birchenough et al., 2015).

Major changes in both the cell-surface and secreted mucins occur in response to intestinal infection (Linden et al., 2008). The integrity of the mucus layer is also related to chronic inflammation of the colon (Paone and Cani, 2020; Lennon et al., 2014). The absence of MUC2 typically results in disruption of the mucus gel structure and are thought to contribute to the pathogenesis of inflammatory bowel diseases (Qiao et al., 2025) and to interactions with the immune system (Pelaseyed et al., 2014).

## Coordination of water and mucus secretion

The tubular lumen of the colon is hydrated and lubricated by the viscoelastic mucus layer(s) which result from coordinated water and mucus secretion; processes which are independently regulated, mechanistically distinct but functionally coupled. These involve the two predominant cell types of the colonic epithelium: colonocytes and goblet cells. Mucus secretion and water/electrolyte secretion are normally coupled at both the cellular and systems level. The coupling is crucial since mucus without hydration is dysfunctional, and water secretion alone without mucus leaves the epithelium exposed. Since extracellular regulators of colonic fluid and mucus secretion are shared (Table 1) and signal transduction pathways in colonocytes and in goblet cells are similar the linkage may be parallel but separate synchronous activation of colonocytes and goblet cells and may be regulated by lamina propria immune cells (Stack et al., 1995) or nerves (Sharkey and Mawe, 2023).

**FIGURE 1:**
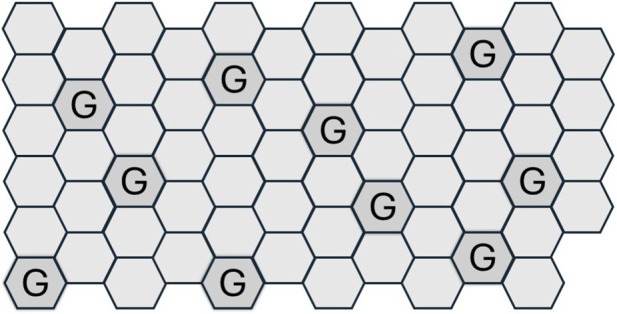
A topographical representation of colonocytes and goblet cells (G) arrange as a simple monolayer in a ratio similar to that observed in human colon. Most of the epithelial cells are colonocytes and goblet cells are the second most abundant. Consequently, mucins and water are secreted from separate but adjacent cell types. Such an arrangement is an efficient one for the generation of a uniform gel.

Two-dimensional representations of epithelial sheets are often cross sectional. In contrast, Figure 1 is a planar depiction or luminal aspect view of the epithelial surface. The proportion of goblet cells relative to enterocytes increases from the proximal to distal intestine and can be up to one-fifth the number of colonocytes (Paone and Cani, 2020; Kim and Ho, 2010; Ermund et al., 2013a; Ma et al., 2018; Konig et al., 2016). Mucin release is often triggered together with fluid secretion. This ensures that the secreted mucus becomes hydrated and spreads across the epithelial surface (McCauley and Guasch, 2015). If mucus and water secretion are coordinately regulated but temporally phased this could account for the formation of the lamellar appearance of native mucus gels.

**FIGURE 2:**
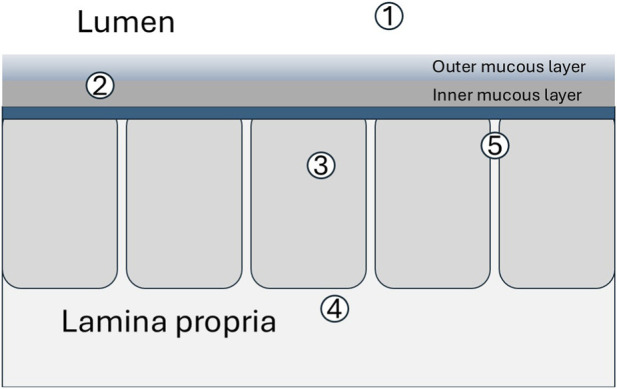
Potential sources of water to hydrate newly extruded mucus include 1. luminal water, 2. water recycled within gel as mucins degrade, 3. intracellular water actively secreted, 4, extracellular fluid absorbed across basolateral domain of epithelial cells (transcellular) and 5. between epithelial cells (paracellular) driven by osmotic gradients.

Mucus is hygroscopic due to its heavily glycosylated mucins as well as secreted electrolytes which exert an osmotic drag for water. Thus water incorporated into the gel may include secreted water and/or luminal water osmotically drawn into the gel as it forms (Figure 2). That secreted water contributes to the native gel is supported by findings that activated chloride secretion, upon which water secretion depends, is important for induced mucus secretion from intestinal epithelial monolayers (Benedetto et al., 2019). Furthermore, mucous gels produced *in vitro* using air-liquid interface mucus secreting monolayers are similar biochemically and rheologically to native gut mucus (Howard et al., 2021).

The colonic microbiome influences both mucus and water secretion by interacting with host cells, influencing mucus production (synthesis, thickness, degradation) and affecting water balance through bacterial metabolites such as short-chain fatty acids (SCFAs) and immune signalling, which contribute to enterocyte function and the gut barrier (Fusco et al., 2023). Thus commensal bacteria stimulate mucus growth and integrity. In dysbiosis microbes can contribute to pathologies including acute diarrhoea (Li et al., 2021; Chung and Le, 2022) as well as being associated with chronic inflammatory bowel diseases (Iliev et al., 2025; Zhang et al., 2026; Shan et al., 2022) including ulcerative colitis (Swirkosz et al., 2023; Ren et al., 2025) which involve perturbations in the colonic microbiome.

## Motility matters

Colonic smooth muscle is responsible for segmental contractions and peristalsis. These are powerful mechanical movements which propel material along the length of the colon and help mixing and short-term storage of contents (Corsetti et al., 2019; Spencer and Hu, 2020; Morales-Soto and Smith-Edwards, 2025). Despite these gross and coordinated cycle of contraction and relaxation the surface mucus remains intact as a gel due to its intrinsic elasticity so that the gel under shear stress behaves like an elastic solid even during churning of smooth muscle. Gross motility and epithelial transport within the colon may be linked (Greenwood and Davison, 1987; Waclawikova et al., 2022). Such linkage may be parallel yet separate synchronous regulation of smooth muscle and epithelial colonocytes. For example, fluid secretion is stimulated by distension (Elfers et al., 2023) and mucus secretion is also stimulated by mechanical movements and shear forces (Jiang et al., 2021). As mucus degrades and is released as a consequence of motility, its components are used by the enteric bacteria as an energy source (Kim and Ho, 2010). The released monosaccharides are converted by bacterial metabolism into short fatty acids which can, in turn, diffuse through the inner mucus layer and provide nutrition to the epithelium.

Motility, like fluid secretion, is regulated by vagal control, the neuroendocrine and enteric nervous systems and by inflammation via the production of neuroactive substances, metabolites, and hormones. Studies indicate that gut microbiotas are capable of producing or stimulating the production of neurotransmitters, including acetylcholine and serotonin, (Yano et al., 2015; Engevik et al., 2021; Loh et al., 2024)^,^ which are also endogenous regulators of gut motility and colonic secretion of water and of mucus, A relatively understudied type of intestinal muscle movement is micromotility which refers to the small-scale movements within the intestine, which occur independently of the larger intestinal wall movements. Such relatively subtle continuous movements may help to extrude the contents of glandular crypts and mix the mucus layer as mucins, water and other components are secreted into the lumen. Micromotility may produce an aggregation phenomenon analogous to that which occurs in ciliated epithelia (Cicuta, 2020). Thus the structured molecular network in the inner lamellar sheet like structure (Wagner et al., 2018) is ideal for intra-gel distribution of products secreted from less numerous colonic epithelial such as antimicrobial substances and IgA (Bansil and Turner, 2018).

## Conclusion

Much research on human gut function has focussed on disease or pathology (Moran and Jackson, 1992). From a physiological perspective, intestinal tissues in isolation were popular for decades as tools of the bioassay which preceded proteomics, molecular biology and ‘omics’ but were hugely significant in basic and applied pharmacology (Bakhle, 2020). Further reductionism using single cells, cocultures and organoids (Flood et al., 2024; Clevers, 2009) has unveiled the significance of specific genes, molecules, and cells (Burclaff et al., 2022) along with the microbiota (Kuziel and Rakoff-Nahoum, 2022; Cresci and Bawden, 2015; Heintz-Buschart and Wilmes, 2018) to understand their roles in gut function and disease but it remains a challenge to understand these dynamic networks at a systems biology level (Cheng et al., 2010).

Planar representations of epithelia as monolayers (Figure 1) offer simplicity of thinking and of experimental modelling. Recent efforts have developed three-dimensional (3D) cell cultures as systems that better mimic *in vivo* physiology (Fang and Eglen, 2017). Human derived spheroids, organoids, scaffolds, hydrogels, organs-on-chips, and 3D bioprinting may replicate tissue architecture, microbiome interfaces, and mechanical forces (like peristalsis or micromotility) (Villegas-Novoa et al., 2024; Sontheimer-Phelps et al., 2020; Shin and Kim, 2022; Beamer et al., 2023). Along with live cell imaging techniques more opportunities than ever before are available to address what we do not yet know about functionally coupled water and mucus secretion (Song et al., 2023). Multiomics approaches to unveiling the gut microbiome, including metatranscriptomics, metaproteomics, and metabolomics (Dey, 2025), may be extended from taxonomy to address specific questions related to integrative physiology and mechanism(s) of disease.

The processes of fluid and mucus secretion in colon are functionally coupled but how this is achieved is unclear. Causation is difficult to demonstrate in biology because biological systems are complex and dynamic. Limitations on current understanding may be met with advances in cell biology (Cleynen and Laukens, 2019), microbiome sequencing (Dey, 2025), imaging and the emerging interest in mucus biology associated with drug delivery (Kramer and Ribbeck, 2023). It ultimately falls to a supradisciplinary systems approach that integrates biology, physics, chemistry, computational modelling, dynamic imaging, and data science to understand the gastrointestinal tract as a complex, kinetic system.
